# Another Way to Break Hearts: Reverse Takotsubo Cardiomyopathy

**DOI:** 10.7759/cureus.24537

**Published:** 2022-04-27

**Authors:** Yixin Zhang, Swetha R Nuthulaganti, Kevin Liu, Anamarys Blanco, Khadeeja Esmail

**Affiliations:** 1 Internal Medicine, University of Florida College of Medicine – Jacksonville, Jacksonville, USA; 2 Cardiology, University of Florida College of Medicine – Jacksonville, Jacksonville, USA; 3 Cardiology, University of Florida College of Medicine - Jacksonville, Jacksonville, USA; 4 Cardiology, University of Florida (UF) Health Jacksonville, Jacksonville, USA

**Keywords:** cardiac imaging-mri, post cardiac arrest care, acute cardiac care, cardiac arrest, reverse takotsubo cardiomyopathy

## Abstract

A 34-year-old female was found to be hypoxic shortly after intubation during elective eye surgery. The patient then went into ventricular fibrillation leading to cardiac arrest. Return of spontaneous circulation (ROSC) was achieved after several rounds of cardiopulmonary resuscitation with epinephrine. The patient was immediately taken for cardiac catherization which revealed angiographically normal coronary arteries. A computed tomography angiogram chest showed pulmonary embolism and unclear chronicity. Transthoracic echocardiogram (TTE) showed a reduced ejection fraction of 30%-35% with nearly akinetic basal walls, consistent with reverse Takotsubo cardiomyopathy. The patient was started on anticoagulation and was successfully extubated shortly afterward. Cardiac magnetic resonance imaging (MRI) one week later revealed a recovered left ventricular ejection fraction. Our case demonstrated variants of Takotsubo cardiomyopathy while highlighting the notion that cardiac function can be temporarily compromised by acute physiological stressors.

## Introduction

Takotsubo is a Japanese word for “octopus pot.” Takotsubo cardiomyopathy (TC) is named after this term as its typical transthoracic echocardiogram (TTE) findings (apical akinesis and ballooning) resemble the narrowed-neck and rounded bottom fishing equipment that the Japanese use to trap octopus [1]. Several variant forms of TC have then been reported with similar clinical presentation and disease course with the exception of the different patient populations. We here, present a reverse form of TC found in a young female.

## Case presentation

**Figure 1:**
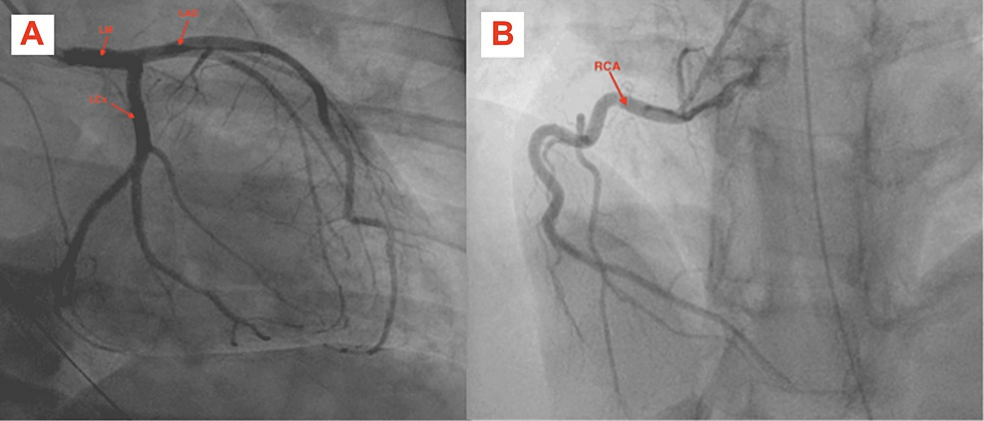
RAO-CAU view showing patent left main, left anterior descending and left circumflex artery (A), and right coronary artery (B).

**Figure 2:**
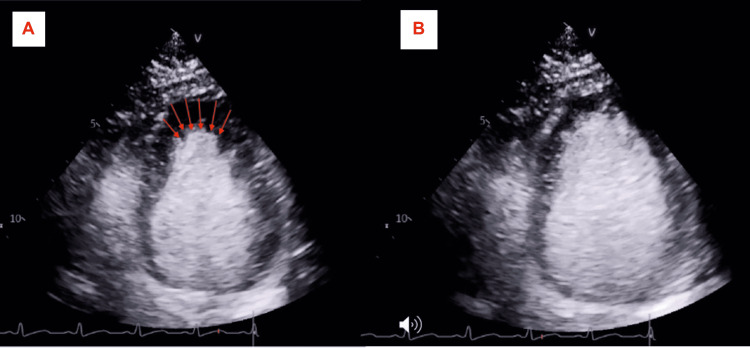
Apical four-chamber view on TTE showing basal akinesis with preserved apical contraction (A, B).

A 34-year-old female with a history of diabetes mellitus type II presented with cardiac arrest. The patient was undergoing elective eye surgery for strabismus but was found to be hypoxic shortly after intubation and induction of anesthesia. She then went into ventricular fibrillation (seen on telemetry, unfortunately, no EKG was obtained during acute resuscitation) leading to cardiac arrest and achieving a return of spontaneous circulation (ROSC) after four rounds of cardiopulmonary resuscitation, including four unsynchronized cardioversion at 120 joules. Afterward, the patient was sent to the emergency room for further management. The initial troponin level was 104 ng/L on a high sensitivity troponin test (reference range <14 ng/L) which then peaked at 1,623 ng/L after three hours. The basic metabolic panel was negative for any significant electrolyte abnormalities. The thyroid panel was within normal limits. Electrocardiogram revealed sinus tachycardia and PR interval shortening with nonspecific T-wave changes. The patient was taken for cardiac catheterization, revealing angiographically normal coronaries arteries (Figures 1A, 1B) and a TTE showed reduced ejection of 30%-35% with basal hypokinesis, consistent with reverse TC (Figures 2A, 2B, Video 1). The patient remained on mechanical ventilation and was able to be extubated 72 hours later without complications. Cardiac magnetic resonance imaging (MRI) one week later showed a recovered left ventricular ejection fraction (LVEF) of 57% with normalization in basal wall contraction (Video 2).

**Video 1:** TTE with contrast showing a hyper-dynamic left ventricular apex and an akinetic left ventricular base.

**Video 2:** Cardiac MRI short-axis view at the level of left ventricular base showing normal contraction of the basal wall and improved ejection fraction.

## Discussion

TC aka broken heart syndrome is an acute left ventricular dysfunction triggered by emotional or physiological distress [2]. Our patient’s cardiac wall motion abnormality cannot be explained by a single epicardial vascular distribution proven by the absence of obstructive angiogram is one of the hallmarks for diagnosis of TC based on the Mayo Clinic Criteria [3]. Several variants of TC demonstrating atypical segments of ventricular wall motion abnormality have been reported in current literature. Our patient was found to have classical features of rTC with apical-sparing and basal akinesis. In a study where 1,750 patients from the International Takotsubo Registry were diagnosed with TTC between 1998 and 2014, only 39 patients (2.2%) have basal wall akinesis [4]. A variety of triggers have been reported as precipitating factors for rTC (Table 1) [2]. While the exact pathophysiology of TC is not fully understood, the leading theory is that a precipitating factor (a non-exhaustive list of examples is shown in Table 1) results in a catecholamine surge as a response to physiological stress. The increased level of catecholamine can promote microvascular dysfunction, coronary vasospasm, and impaired fatty acid metabolism, resulting in regional myocardial stunning and injury [1,5,6].

**Table 1:** List of precipitating factors that were previously reported in the literature.

Precipitating factors for Reverse Takotsubo Cardiomyopathy
Estrogen deficiency
Intracranial hemorrhage
General Anesthesia
Eating disorders
Multiple Sclerosis
Serotonin syndrome
Black widow spider bites
Yohimbine use

In addition, the age group for rTC is often younger compared to typical TC, with a mean age of 36 [2,4,5]. The proposed theory is that premenopausal women have their highest density of catecholamine receptors within the base of the heart (in comparison with postmenopausal women whose catecholamine receptors are more concentrated at the apex of the heart), which explains why rTC occurs more frequently in younger women [5]. Another unique clinical finding for rTC patients is that they often have higher serum cardiac biomarkers than apical TC patients. This finding is likely because the basal myocardium has relatively more tissues, so more myocardial injury is involved [5].

What ultimately caused our patient’s transient cardiomyopathy remains polyfactorial. There have only been a few scattered case reports with general anesthesia-induced rTC, but not enough data supporting any specific anesthetic agent as a strong associator. The triggering events for our patient perhaps are likely a combination of general anesthesia and hypoxia causing severe physiological distress. it is very difficult to contribute one signal stressor as the sole cause. Furthermore, rTC tends to have a lower ejection fraction at presentation compared to typical TC with a paradoxical faster recovery period [7]. Our patient’s cardiac function normalized within one week once her stressors were removed, demonstrated by her cardiac MRI.

## Conclusions

The primary focus in our case is to show that cardiac function can be directly affected by acute physiological stressors resulting in rTC that is often found in a different patient profile compared to the classical TC. It is important to recognize disease variants for TC with its atypical TTE findings. Awareness of this variant can facilitate its prompt recognition in the correct clinical scenario, leading to a correct treatment plan.
